# Supplementary material for: Fezf2 regulates differentiation of Aire-expressing and post-Aire mimetic epithelial populations maintaining thymic homeostasis
Source: bioRxiv. 2026 Jan 1:2025.12.31.697099. Preprint. [Version 1] doi: 10.64898/2025.12.31.697099 (PMC12776557; doi:10.64898/2025.12.31.697099)
Supplement: 1 [file NIHPP2025.12.31.697099V1-supplement-1.pdf]

bioRxiv preprint doi: https://doi.org/10.64898/2025.12.31.697099; this version posted January 1, 2026. The copyright holder for this preprint (which was not certified by peer review) is the author/funder, who has granted bioRxiv a license to display the preprint in perpetuity. It is made available under aCC-BY 4.0 International license.

Supplementary Information:

Supplementary Tables

Table S1. Immature mTEC subset highly differentially expressed genes in WT mTECs. Highly differentially expressed genes for each immature mTEC subcluster in WT mTECs were calculated using scvi-tools, identified as genes with a bayes factor greater than 3, a mean log-fold change between the population of interest and all other WT mTECs greater than 1 or less than -1, and expression in at least 10% of the immature mTEC subcluster.

Table S2. Flow cytometry marker genes used for mTEC subpopulation identification.

mTEC subsets	Flow cytometry markers	Immunofluorescent markers
**Common TECs**	EpCAM	K5 and K8
**cTECs**	Ly51	NA
**Immature TECs**	Ccl21	Ccl21
**Aire+ mTECs**	Aire and MHC II	Aire
**M cells**	Gp2	Gp2
**Neuroendocrine cells**	CD177	NA
**Tuft cells**	Dclk1	Dclk1

Table S3. WT vs Aire KO highly differentially expressed genes. Highly differentially expressed genes between WT and Aire KO samples for each cell type were calculated using scvi-tools, identified as genes with a bayes factor greater than 2.5, a mean log-fold change between WT and Aire KO cells greater than 1 or less than -1, and expression in at least 5% of either sample while also excluding genes with low maximum expression. differentially expressed genes were filtered on genes that were consistent across both Aire KO replicates and not differentially expressed between WT or KO samples in any cell type.

Table S4. WT vs Fezf2 cKO highly differentially expressed genes. Highly differentially expressed genes between WT and Fezf2 cKO samples for each cell type were calculated using scvi-tools, identified as genes with a bayes factor greater than 2.5, a mean log-fold change between WT and Fezf2 cKO cells greater than 1 or less than -1, and expression in at least 5% of either sample while also excluding genes with low maximum expression. differentially expressed genes were filtered on genes that were consistent across both Fezf2 cKO replicates and not differentially expressed between WT or KO samples in any cell type.

Table S5. TRA analysis of genes in the cellxgene scRNA-seq atlas. The expression of each gene in the cellxgene scRNA-seq atlas was averaged per cell type, and the resulting mean expression distribution across cell types was used to compute Tau and Shannon entropy scores per gene for TRA classification (**see methods**).

bioRxiv preprint doi: https://doi.org/10.64898/2025.12.31.697099; this version posted January 1, 2026. The copyright holder for this preprint (which was not certified by peer review) is the author/funder, who has granted bioRxiv a license to display the preprint in perpetuity. It is made available under aCC-BY 4.0 International license.

Supplementary Figures

Figure S1

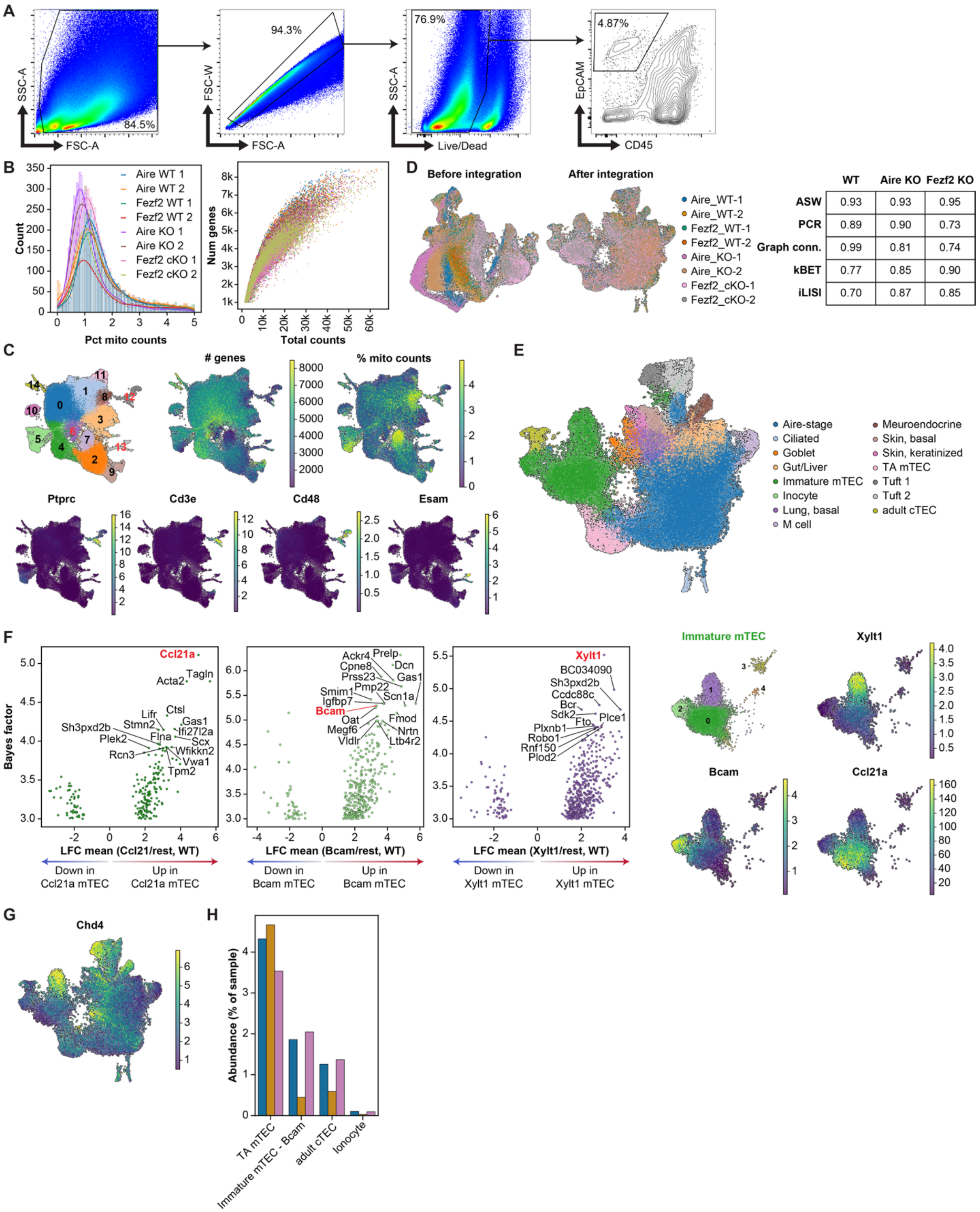

bioRxiv preprint doi: https://doi.org/10.64898/2025.12.31.697099; this version posted January 1, 2026. The copyright holder for this preprint (which was not certified by peer review) is the author/funder, who has granted bioRxiv a license to display the preprint in perpetuity. It is made available under aCC-BY 4.0 International license.

Figure S1. Collection and annotation of WT, Fezf2 cKO and Aire KO single-cell RNA-seq datasets. (**A.**) Sorting strategy of EpCAM+ CD45- TECs for 10X scRNA-seq. (**B.**) Per-cell mitochondrial read percentage (left) and total counts versus number of genes detected (right) colored by batch for 4-6-week-old WT, Aire KO, and Fezf2 cKO EpCAM+ CD45- TECs scRNA-seq datasets. (**C.**) Feature plots depicting the cluster number, number of detected genes, percent mitochondrial reads, and normalized expression for non-TEC markers. Cluster numbers in red were non-TECs removed from downstream analysis. (**D.**) Feature plots colored by sample before and after batch correction. Table depicts integration QC metrics calculated using scib scaled to range from 0 (poor integration) to 1 (well-integrated) (44). (**E.**) Predicted cell type labels of individual cells determined using celltypist reference label transfer from a published mTEC scRNA-seq dataset sampling mimetic cells with high resolutions (9). (**F.**) Differentially expressed genes analysis (left) and feature plots of normalized expression for marker genes (right) used to identify and reannotate immature mTEC subclusters. (**G.**) Normalized expression of Chd4 in WT TECs. (**H.**) Fraction of cells annotated as each TEC subpopulation across the WT, Aire KO, and Fezf2 cKO samples for subpopulations with a mean log2(fold change) between WT and Fezf2 cKO samples less than 0.5 or greater than -0.5. Colored bar depicts the mean abundances across 2 (Fezf2 cKO and Aire KO) or 4 (WT) independent replicates.

bioRxiv preprint doi: https://doi.org/10.64898/2025.12.31.697099; this version posted January 1, 2026. The copyright holder for this preprint (which was not certified by peer review) is the author/funder, who has granted bioRxiv a license to display the preprint in perpetuity. It is made available under aCC-BY 4.0 International license.

Figure S2

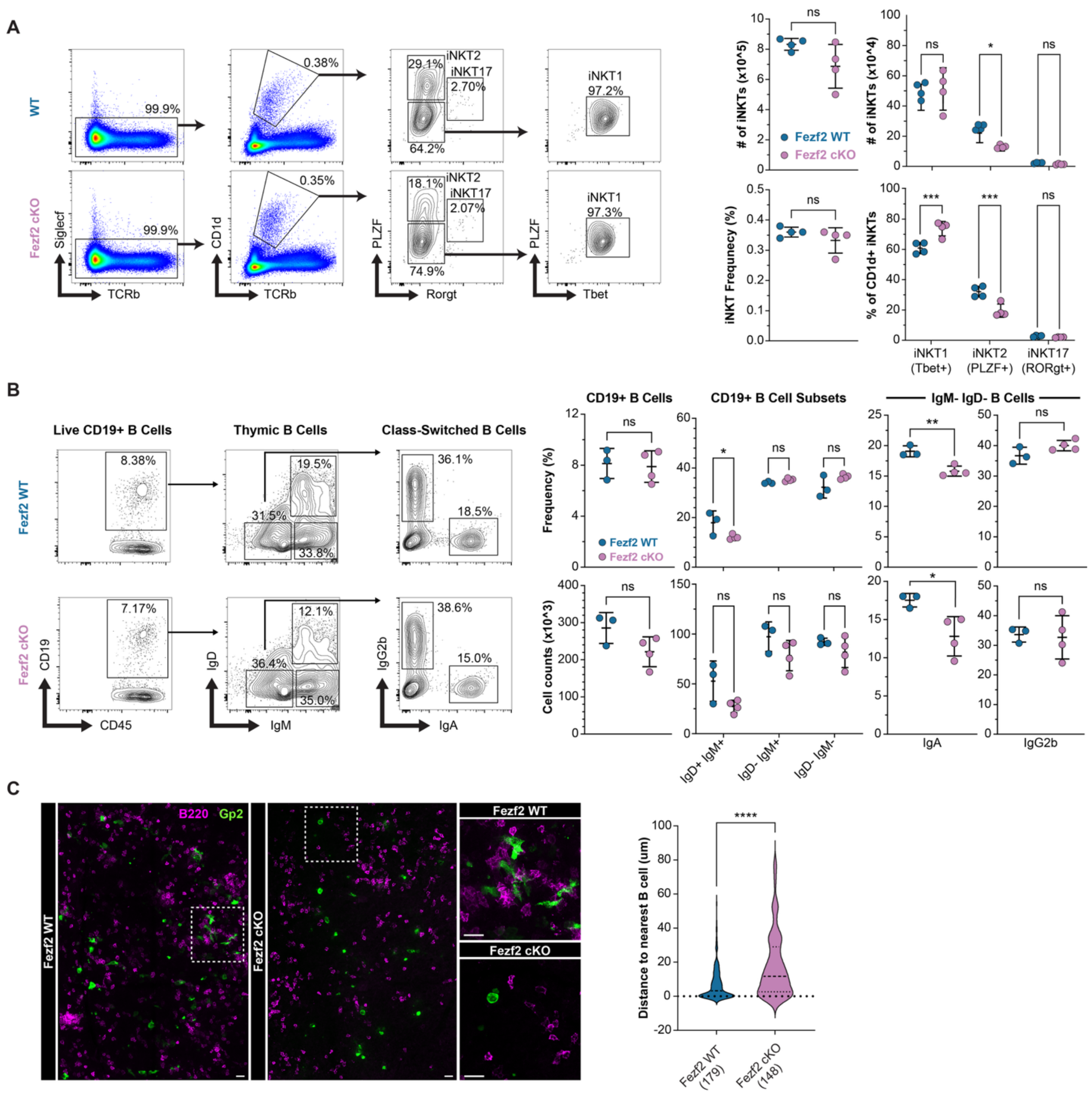

bioRxiv preprint doi: https://doi.org/10.64898/2025.12.31.697099; this version posted January 1, 2026. The copyright holder for this preprint (which was not certified by peer review) is the author/funder, who has granted bioRxiv a license to display the preprint in perpetuity. It is made available under aCC-BY 4.0 International license.

Figure S2. Developmental defects in Fezf2 cKO thymus disrupt stromal-immune crosstalk. (**A.**) Representative FACS plots of each population (left) along with frequency or absolute number of iNKTs (middle) and iNKT subsets (right) in 6-week-old WT (n=4) and Fezf2 cKO (n=4) mice. (**B.**) Representative FACS plots (left) and frequency or total counts (right) of thymic B cell subsets in 6-week-old WT (n=4) and Fezf2 cKO (n=4) mice. (**C.**) Representative immunofluorescent staining of Gp2 and B220 in 6-week-old WT and Fezf2 cKO thymi (left) and quantification of the distance between Gp2+ mTECs and the nearest B cell (right) (n=3). Scale bars are 20 um (C), and 10 um (C, inserts). Statistical significance was calculated using unpaired Student's t test (A-B).

bioRxiv preprint doi: https://doi.org/10.64898/2025.12.31.697099; this version posted January 1, 2026. The copyright holder for this preprint (which was not certified by peer review) is the author/funder, who has granted bioRxiv a license to display the preprint in perpetuity. It is made available under aCC-BY 4.0 International license.

Figure S3

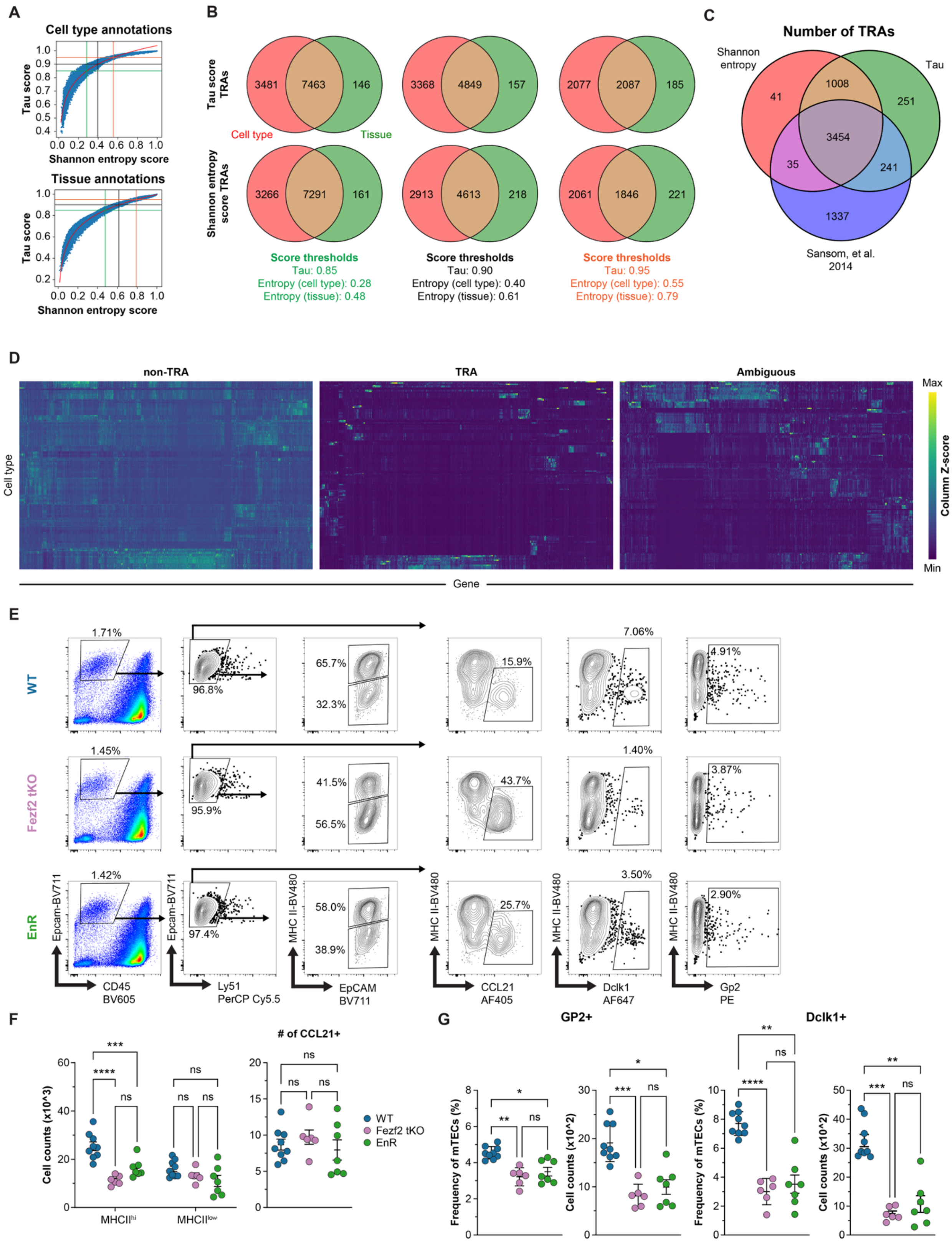

bioRxiv preprint doi: https://doi.org/10.64898/2025.12.31.697099; this version posted January 1, 2026. The copyright holder for this preprint (which was not certified by peer review) is the author/funder, who has granted bioRxiv a license to display the preprint in perpetuity. It is made available under aCC-BY 4.0 International license.

Figure S3. Identifying TRAs using the cellxgene scRNA-seq atlas.
(**A.**) Shannon entropy vs Tau scores generated from cell type and tissue annotations in the cellxgene atlas (49). Vertical and horizontal lines depict score thresholds used for TRA analyses. Red line shows a log2 model fit to determine the appropriate Shannon entropy score for each Tau threshold. (**B.**) Overlap in TRA gene lists between cell type- and tissue-level cellxgene atlas scoring. (**C.**) Overlap between TRAs from Tau and Shannon entropy scoring of cellxgene cell types and a previously published TRA list (48). (**D.**) Cell type expression in the cellxgene atlas of TRAs/non-TRAs identified by both scoring metrics (left, middle) or by one scoring metric (right). (**E.**) Representative flow cytometry gating strategy for mTEC subpopulations across WT, Fezf2 tKO, and Fezf2 tKO EnR mice. (**F.**) Absolute number of major mTEC subpopulations across 4- to 7-week-old WT, Fezf2 tKO and Fezf2 tKO EnR mice. (n=6-9 mice). (**G.**) Frequency and absolute number of mimetic cell populations across 4- to 7-week-old WT, Fezf2 tKO and Fezf2 tKO EnR mice. (n=6-9 mice). Statistical significance was calculated using two-way ANOVA (Sidak’s test) with multiple comparisons (F-G).

bioRxiv preprint doi: https://doi.org/10.64898/2025.12.31.697099; this version posted January 1, 2026. The copyright holder for this preprint (which was not certified by peer review) is the author/funder, who has granted bioRxiv a license to display the preprint in perpetuity. It is made available under aCC-BY 4.0 International license.

Figure S4

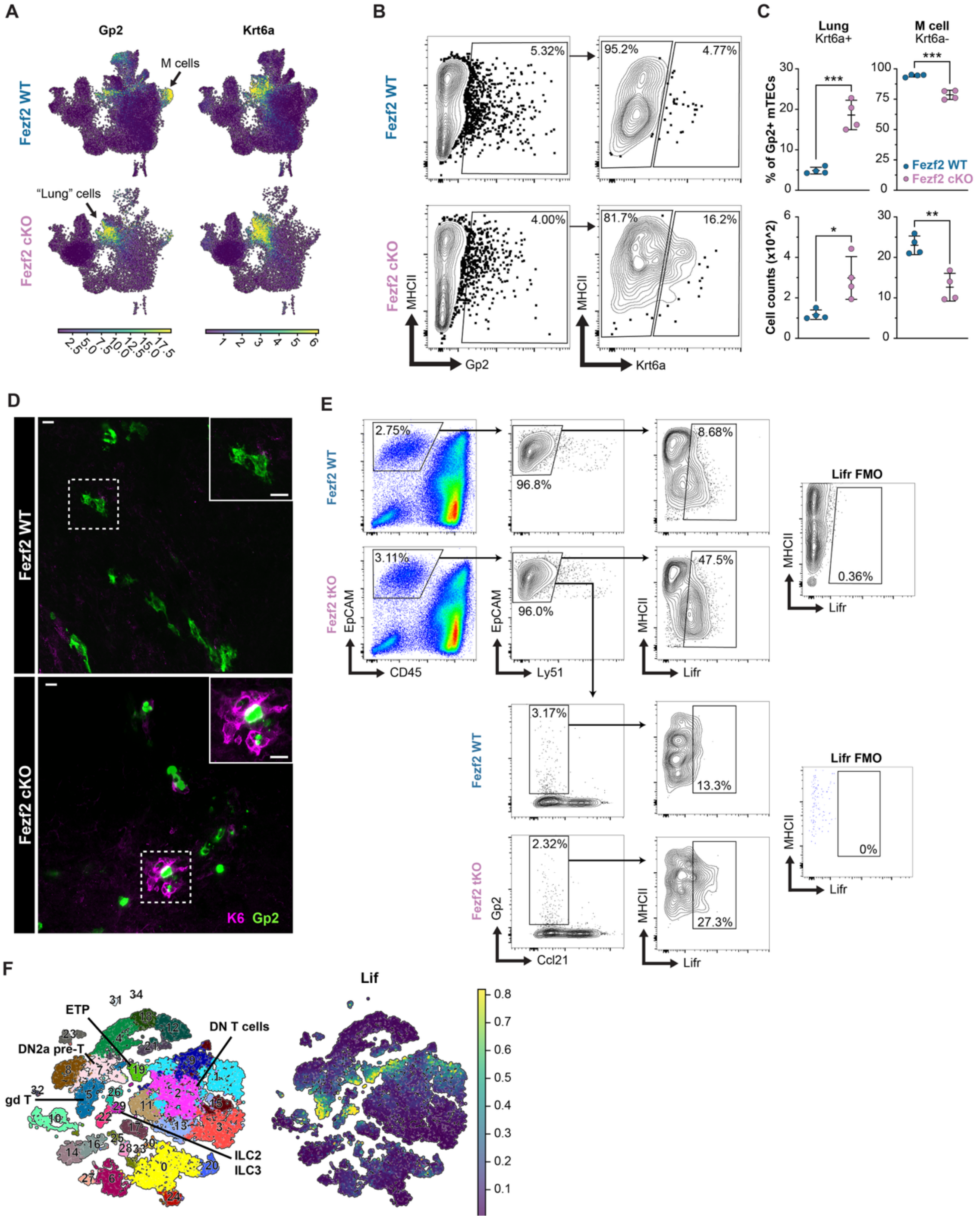

1
2

bioRxiv preprint doi: https://doi.org/10.64898/2025.12.31.697099; this version posted January 1, 2026. The copyright holder for this preprint (which was not certified by peer review) is the author/funder, who has granted bioRxiv a license to display the preprint in perpetuity. It is made available under aCC-BY 4.0 International license.

Figure S4. Lifr expression in the murine thymus.
(**A.**) Feature plots of normalized Gp2 and Krt6a expression in WT and Fezf2 cKO TECs. (**B.**) Representative flow cytometry gating for basal lung/goblet (Krt6a+Gp2+) and microfold (Krt6a-Gp2+) mTECs. (**C.**) Quantification of frequency and count of basal lung/goblet and microfold mimetic cells in 6-week-old WT (n=4) and Fezf2 cKO (n=4) thymi by flow cytometry. (**D.**) Immunofluorescent staining of Gp2 and Krt6a in WT and Fezf2 cKO mice. Scale bars are 50um. (**E.**) Representative flow cytometry gating used to identify Lifr-expressing mTECs. (**F.**) Lif expression in hematopoietic cells from the murine thymus before and after Dex treatment (31).
